# Patient influencing doctor-patient dispute trials: evidence from China

**DOI:** 10.3389/fpsyg.2026.1734171

**Published:** 2026-01-30

**Authors:** Jiangjie Sun, Shuxing Shen, Qinglin Han, Liping Zhang

**Affiliations:** 1 School of Management, Hefei University of Technology, Hefei, Anhui, China; 2 School of Health Care Management, Anhui Medical University, Hefei, Anhui, China; 3 School of Marxism, Anhui Medical University, Hefei, Anhui, China

**Keywords:** doctor-patient disputes, doctor-patient relationship, empirical research, hospital economic compensation, patient factors

## Abstract

**Background:**

Doctor-patient dispute prevention strategy has become a hot issue in the field of doctor-patient relationship risk management.

**Objective:**

To clarify the impact of patient-related factors on the levels of economic compensation awarded to hospitals in medical disputes and on judicial outcomes, thereby providing empirical evidence for optimizing risk management strategies in medical disputes and enhancing the efficiency of judicial proceedings.

**Methods:**

A convenience sample of 863 cases was selected, and the data were processed using SPSS 26.0. A comparison of characteristic variables was conducted to examine differences in patient-level hospital economic compensation ratings and dispute trials outcomes. Correlation analysis was performed between dependent and independent variables. Based on these results, a binary/ordinal regression analysis was conducted on the hospital economic compensation ratings / dispute trials outcomes for patients’ health losses.

**Results:**

The hospital economic compensation ratings differed significantly by dispute initiator and disability ratings (*Ps < 0.001*). The hospital economic compensation ratings was positively correlated with dispute trials outcomes, dispute initiator, and doctor-patient relationship risk attributes (*Ps < 0.05*) and negatively correlated with health damage disability ratings (*p < 0.01*). The investigated patient factors were “not significantly associated” with dispute trials outcomes in this study (*Ps > 0.05*). Regression analysis revealed that the disability level of injury and the doctor-patient relationship risk attributes has a significant negative impact on the amount of hospital economic compensation (*β = −0.225 & −0.644, p ≤ 0.007*).

**Conclusion:**

The patient’s health damage disability ratings and doctor-patient relationship risk attributes directly influenced the hospital economic compensation ratings, while the initiator of the dispute and the patient’s gender had indirect influences. It is recommended to optimize risk management in medical disputes by strengthening communication between doctors and patients, enhancing mutual trust, and standardizing guidance for patients exercising their rights.

## 1 Introduction

The relationship between doctors and patients is essential for providing high-quality healthcare services (Zhang et al., 2019). Especially in China, influenced by 5,000 years of Chinese civilization and traditional culture, a positive doctor-patient relationship has become a primary resource for hospitals to attract patients (Lowry et al., 2010). Therefore, building a high-quality doctor-patient relationship has become a top priority for Chinese healthcare practitioners.

Globally, medical disputes and the resulting lawsuits and compensation claims pose significant challenges to healthcare systems. According to incomplete statistics, the United States spends approximately $55 billion annually to address medical disputes, with 42.2% of physicians facing lawsuits during their careers (Li, 2013; Pepper and Slabbert, 2011), which has become a major impediment to the development of healthcare in the U.S. In the UK, 63% of family doctors have experienced violence (Khan et al., 2010), and the actual rate is even higher because of the possibility of under-reporting of violence. Analysis of the UK National Health Service Dispute Trials Authority database found that wrist medical malpractice accounted for 48% of all medical malpractice claims from 1994 to 2001 (Khan and Giddins, 2010; DeNoble et al., 2014). Another study evaluating data on wrist medical malpractice claims in the UK National Health System from 2000 to 2006 found that the average amount of damages for wrist cases was $86,089 (Atrey et al., 2010). Other medical claims were not included in the statistics, which highlights the significant financial burden of medical malpractice on the development of the NHS in the UK. In China, as public awareness of rights grows, medical disputes have garnered widespread attention.

## 2 Theoretical background and research hypothesis

### 2.1 Research on doctor-patient disputes

Existing research on medical disputes has predominantly focused on physician-related factors, primarily encompassing the definition of defensive medicine’s scope and boundaries (Tancredi and Ba, 1978; Hao et al., 2022; Kapp, 2016; Raposo, 2019; Garattini and Padula, 2020), studies on its current status and causes (Tan, 2011; Borgan et al., 2020; Wang et al., 2022; Du and Hou, 2021), investigations into the behavioral characteristics of practitioners engaging in defensive medicine (Unal and Akbolat, 2022; Delpini and Russu, 2022; Williams et al., 2021; Ortashi et al., 2013), and governance approaches for defensive medical practices (Jingwei, 2014; Catino and Celotti, 2009; Hermer and Brody, 2010). Exploration of patient-related factors remains relatively scarce. Although existing research suggests patients may influence doctor-patient relationships through defensive behaviors (Finocchiaro et al., 2011), no systematic conclusions have been reached regarding how patient characteristics specifically impact the core outcomes of dispute cases—hospital economic compensation and judicial rulings. Furthermore, as the ultimate resolution pathway for medical disputes, the fairness of judicial adjudication remains unsupported by empirical data regarding potential interference from patient-side subjective factors such as disruptive behavior or dispute initiation motives (Li et al., 2024; Sun et al., 2023; Sun et al., 2021). Considering that the risks in doctor-patient relationships primarily refer to the potential for conflicts to arise between physicians and patients, this is a dualistic relationship complicated by interfering factors such as hospitals and illnesses. Simultaneously, physicians are positioned as victims in medical disputes, lacking the motivation to initiate such conflicts. We contend that patients are the dominant actors in medical-patient relationship risks, directly determining their trajectory. Dispute trials, as the statutory avenue for resolving disputes, suffer from drawbacks including time and energy consumption for both parties, complex adjudication procedures, and high costs (Li et al., 2024). How can judicial efficiency be enhanced? From the patient’s perspective, identifying potential factors influencing hospital financial compensation and dispute trials outcomes becomes a critical step. The findings of this study facilitate targeted doctor-patient communication by physicians, thereby improving medical-patient relationships.

The uncertainty of economic expenditure based on patient’s disease treatment and its health benefits may trigger discord in the doctor-patient relationship. In the case of patients with a high degree of disability and damage, or even death, it will involve the choice motivation of health stakeholders (is it to know the truth about the disease treatment or to pursue financial compensation?). The clinical manifestations of the same disease vary among patients, potentially leading to differing treatment outcomes. Individual patient differences create distinct caregiving experiences for health stakeholders, which may result in divergent perceptions of the patient’s recovery outcomes. In general, patients initiate disputes primarily to understand the truth about their diagnosis and treatment, while health stakeholders are more likely to focus on seeking compensation for economic losses. Therefore, the following hypothesis is proposed.

*H1*: Hospital economic compensation levels correlate with the identity of the dispute initiator (patient themselves/non-patient).

The inherent complexity of diseases and the unpredictability of their progression, particularly in patients with acute, critical, and severe conditions, mean that medical risks may unpredictably occur at any time. At the same time, the patient’s disability damage level is directly related to the subsequent quality of life and health service inputs, thus directly affecting the patient’s subsequent health economic expenditures and the standard of living of health stakeholders. Therefore, the following hypothesis is proposed.

*H2*: Hospital economic compensation levels negatively correlate with the disability level of health impairment; higher disability levels indicate lesser health damage.

Based on the impartiality of justice (Sun et al., 2023; Sun et al., 2021), trial outcomes in doctor-patient disputes should not be affected by the patient’s medical behavior, health status, or subjective experiences. Therefore, the following hypotheses are proposed.

*H3*: The level of hospital economic compensation is unrelated to the risk attributes of the doctor-patient relationship (i.e., whether accompanied by disruptive behavior);

*H4*: The trial outcome (win/loss) is unrelated to the dispute initiator, the patient’s gender, the patient’s disability level, or the risk attributes of the doctor-patient relationship.

## 3 Objects and methods

### 3.1 Objects

The research data originates from China’s most authoritative and comprehensive Judgment Document Network, selecting adjudicated medical malpractice cases from 2001 to 2023. The total sample size comprises 4,148 documents, accumulating approximately 29,036,000 characters of textual data. In China, the impact of the 2010 Tort Liability Law on medical-patient relationship risks spanned eleven years until the Civil Code officially took effect on January 1, 2021, at which point it began influencing the adjudication of medical disputes. Following consultation with judicial experts, the research team adopted the Civil Code’s implementation as a dividing point: for data prior to 2020, the entire dataset of rulings from 2001 to 2013 was conveniently selected; To reflect judicial practices following the implementation of the Civil Code from 2021 to 2025, and drawing on the practical experience of our professional legal team, we selected all risk data from 2023, which covers the possible situations of medical-patient relationship risk cases adjudicated since the Civil Code took effect,. This approach reduces noise caused by seasonal variations in disease risks inherent in random sampling, ensuring a degree of representativeness. Ultimately, 863 valid samples were screened based on inclusion and exclusion criteria.

Inclusion criteria:

1. Dispute parties were hospitals and patients/their health stakeholders;
2. Judgment documents were complete with no missing key information (e.g., plaintiff/defendant details, claims, disability assessment opinions, judgment outcomes, and compensation breakdowns);
3. Case nature involved disputes arising from medical treatment during healthcare service delivery.

Exclusion rules:

1. Key information is incomplete (e.g., no explicit disability rating or specific hospital economic compensation);
2. Core dispute centered on the attribution of medical liability assessment rights rather than damages arising from the medical treatment itself;
3. Involvement of third parties (e.g., medical device companies, blood banks) in the dispute, rather than purely doctor-patient conflicts.

### 3.2 Variable assignment and measurement

The core variables and their operational definitions in this study are as follows:

1. Dependent variable:
  Hospital economic compensation refers to monetary compensation paid by hospitals to patients and their families due to medical disputes (unit: yuan). Its grading is based on Articles 1,179 and 1,218 of the Civil Code of the People’s Republic of China, and Articles 12, 15, and 17 of the Interpretation of the Supreme People’s Court on Several Issues Concerning the Application of Law in the Trial of Cases Involving Compensation for Personal Injury. The classification levels are determined by reference to the common distribution of hospital economic compensations: minor injury (≤ 20,000 ¥), general disability (100,000–500,000 ¥), severe disability/death (≥ 1,000,000 ¥).
  The second dependent variable, dispute trials outcome, is a binary variable: partial or full support of the patient representative’s claims is recorded as a win, while full dismissal of claims is recorded as a loss.
2. Independent variables:
  1. Dispute Initiator: Refers to the entity directly initiating the medical-patient relationship dispute, including the patient themselves, immediate family members, legal representatives, and other health stakeholders;
  2. Patient disability ratings: Referenced from the Chinese Classification of Disability ratings for Human Injury (GB/T 31147–2014), categorized as: - Death (most severe impairment) - ratings 1 disability (severe impairment) - ratings 2–3 disability (significant impairment) - ratings 4–5 disability (moderate impairment) - ratings 6 and above disability (mild impairment). Higher disability ratings indicate lesser health impairment severity.
  3. Risk Attribute of Physician-Patient Relationship: In this study, “medical disturbance” refers to acts explicitly documented in court judgments that meet the relevant definitions under the Public Security Administration Punishment Law of the People’s Republic of China or the Regulations on the Prevention and Handling of Medical Disputes. These acts include, but are not limited to: assaulting or verbally abusing medical personnel, intentionally damaging property, setting up memorial altars or placing wreaths, and other acts that severely disrupt medical order. It also includes “acts of disrupting medical order where the judgment document records that the patient or their family only accepts financial compensation from the hospital and refuses to undergo judicial health damage assessment.” The specific categories of conduct, manifestations of conduct, and legal basis are shown in Table 1.

**Table 1:** Categories of medical disturbance behavior, manifestations, and legal basis.

Behavior category	Behavioral performance	Legal basis
Disorderly conduct	Setting up unauthorized memorial altars, displaying funeral wreaths, and illegally storing bodies within medical institutions; hanging banners, blocking entrances, and gathering crowds to cause disturbances, thereby disrupting medical services.	Article 23 of the Public Security Administration Punishment Law; Article 290 of the Criminal Law (Crime of Gathering a Crowd to Disrupt Social Order).
Violent crimes	Assaulting or intentionally injuring medical personnel; Intentionally damaging public or private property of medical institutions.	Articles 43 and 49 of the Public Security Administration Punishment Law; Articles 234 and 275 of the Criminal Law.
Violations of personal rights	Openly insulting, intimidating, or defaming medical personnel; Illegally restricting the personal freedom of medical personnel.	Articles 40 and 42 of the Public Security Administration Punishment Law; Articles 246 and 238 of the Criminal Law.
Other illegal categories	Illegally carrying firearms, ammunition, controlled devices, or hazardous materials into medical institutions; stealing or seizing medical records; gathering crowds to obstruct traffic.	Articles 30, 32, and 49 of the Public Security Administration Punishment Law

If the judgment explicitly records that the patient or their family members committed any one or more of the acts listed in the table above, it shall be coded as 1 = “medical disturbance.” This coding is based on the legal nature of the act itself and does not require the document to mention “police dispatch.” All other cases shall be coded as 2 = “non-medical disturbance.” For more effective data analysis, the detailed assignment of key feature variables is shown in Table 2.

**Table 2:** Risk data characterization variables assignment.

Variant	Categorization	Assignment	Variant	Categorization	Assignment
Dispute initiator	Patient himself	1	Doctor-patient relationship risk attributes	Medical disturbance cases	1
	Not the patient himself	2		Non-medical disturbance cases	2
Dispute trials outcomes	Win	1	Level of hospital economic compensation (Unit: 10, 000 ¥)	(0–2)	1
	Fail	0		[2, 10)	2
Patient health impairment disability ratings	Death	1	Patients’ gender	[10, 50)	3
	First-degree disability	2		[50, 100)	4
	Second- to third-degree disability	3		[100, ∞)	5
	Fourth to fifth ratings disability	4		Male	1
	Level six or above disability	5		Female	2

### 3.3 Statistical methods

Data were processed using SPSS 26.0 software. Descriptive statistical analysis was conducted on the demographic characteristics of the sample, case features, and all variables. The chi-square test was used to analyze differences in economic compensation levels and dispute trials outcomes across respective variables. Spearman’s correlation analysis assessed the strength of relationships between variables. An ordered logistic regression model was applied for the ordered dependent variable (hospital economic compensation levels), while a binary logistic regression model was used for the binary dependent variable (dispute trials outcomes). Control variables were included in both models to account for confounding effects. The ordered logistic regression model passed the Test of Parallel Lines (*p > 0.05*), while the binary logistic regression model passed the Hosmer-Lemeshow test (*p > 0.05*). Pseudo R-squared values were reported for each model to assess explanatory power, indicating good model fit. The two-tailed significance level was set at *α = 0.05*.

## 4 Result

### 4.1 Status of the hospital economic compensation level and dispute trials outcomes

The authors analyzed the differences in case compensation status and dispute trials outcomes by patient gender, disability status, place of residence, and doctor-patient relationship risk attributes. The results are presented in Table 3.

**Table 3:** Differences in hospital compensation levels and dispute trials outcomes from patient side.

Variables		Hospital economic compensation level					Dispute trials outcomes	
		(0–2) (%)	[2, 10) (%)	[10, 50) (%)	[50,100) (%)	[100,∞) (%)	Win a court case (%)	Lose a lawsuit (%)
Disputes initiator	The patient himself	156 (54.2)	14 (52.6)	99 (40.4)	12 (25)	4 (40)	317 (47.2)	97 (50.5)
	non-person	132 (45.8)	129 (47.4)	146 (59.6)	36 (75)	6 (60)	354 (52.8)	95 (49.5)
	*x* ^2^	22.755					0.643	
	*p*	0.000					0.423	
Patient genders	Male	152 (52.8)	124 (45.6)	132 (53.9)	27 (56.2)	7 (70)	338 (50.4)	105 (54.7)
	FEMALE	136 (47.2)	148 (54.4)	113 (46.1)	21 (43.8)	3 (30)	333 (49.6)	87 (45.3)
	*x* ^2^	6.323					1.113	
	*p*	0.176					0.292	
Patient health impairment disability ratings	Death	97 (33.7)	96 (35.3)	117 (47.8)	30 (62.5)	2 (20)	266 (39.6)	76 (39.6)
	First-degree disability	15 (5.2)	15 (5.5)	12 (4.9)	8 (16.7)	4 (40)	43 (6.4)	11 (5.7)
	Second- to third-degree disability	12 (4.2)	16 (5.9)	17 (6.9)	4 (8.3)	2 (20)	41 (6.1)	10 (5.2)
	Fourth to fifth ratings Disability	10 (3.5)	6 (2.2)	13 (5.3)	4 (8.3)	1 (10)	27 (4.0)	7 (3.6)
	ratings six and above	154 (53.5)	139 (51.1)	86 (35.5)	2 (4.2)	1 (10)	294 (43.8)	88 (45.8)
	*x* ^2^	89.981					0.507	
	*p*	0.000					0.973	
Doctor-patient relationships risk attributes	Medical disturbance cases	30 (10.4)	24 (8.8)	11 (4.5)	2 (4.2)	1 (10)	52 (7.7)	16 (8.3)
	Non-medical disturbance cases	258 (89.6)	248 (91.2)	234 (95.5)	46 (95.8)	9 (90)	619 (92.3)	176 (91.7)
	*x* ^2^	7.740					0.070	
	*p*	0.102					0.791	

To facilitate data processing, missing gender is considered equally for both male and female frequencies. When the frequency of missing gender for individual variables is odd in the processing of dispute trials outcomes, it is for the balance of data volume (cross-setting of gender, and 1 more male than female).

Table 3 shows that the distribution of hospital economic compensation level exhibits significant differences based on dispute initiator (*Ps <0.001*) and patient disability ratings (*Ps<0.001*). Specifically, disputes initiated by health stakeholders more frequently resulted in higher hospital economic compensation (≥500,000 yuan); compensation tended to increase with lower disability ratings (i.e., more severe harm). The distribution of hospital economic compensation levels showed no statistically significant differences based on patient gender or risk attributes of the doctor-patient relationship. Furthermore, differences in patient dispute trials outcomes (win rate) were not significant across all patient characteristic variables (Ps > 0.05), preliminarily supporting H5.

### 4.2 Correlations between hospital economic compensation level, dispute trials outcomes, and variables

To clarify the correlation between the hospital economic compensation levels, dispute trials outcomes, and variables, the author conducted an analysis on the correlation of these variables. The results are shown in Table 4.

**Table 4:** The correlation between hospital economic compensation levels and judicial outcomes at the patient level with various variables.

	Hospital economic compensation level	Dispute trials outcomes	Dispute initiator	Gender	Patient health impairment disability ratings	Risk attributes of doctor-patient relationship
Hospital economic compensation level	1					
Dispute trials outcomes	0.580^**^	1				
Dispute initiator	0.146^**^	0.027	1			
Gender	−0.025	0.043	−0.105^**^	1		
Patient health impairment disability ratings	−0.200^**^	−0.010	−0.804^**^		1	
Risk attributes of doctor-patient relationship	0.082^*^	0.009	0.038		−0.012	1

The higher the disability rating, the less severe the injury *p < 0.05; **p < 0.01.

Table 4 indicates that the hospital economic compensation level positively correlates with dispute trials outcomes (*r = 0.580, p < 0.01*) and dispute initiators (*r = 0.146, p < 0.05*), while negatively correlating with patient health impairment disability ratings (*r = −0.200, p < 0.01*). And a weak positive correlation with the risk attributes of the doctor-patient relationship (*r = 0.082, p < 0.05*), confirming Hypotheses 1, 2, and 3. The correlations between dispute trials outcomes and other variables were all statistically insignificant (*Ps>0.05*), further supporting H4. Additionally, the dispute initiator showed negative correlations with patient gender (*r = −0.105, p < 0.01*) and disability level due to health impairment (*r = −0.804, Ps < 0.01*). Patient gender was positively correlated with disability level due to health impairment (*r = 0.107, Ps < 0.01*).

### 4.3 Influencing factors of hospital economic compensation level and dispute trials outcomes

To identify the factors influencing hospital economic compensation levels, an ordered logistic regression analysis was conducted with hospital economic compensation level as the dependent variable, incorporating all patient-related independent variables and covariates. The result of Test of Parallel Lines (*p > 0.05*) indicates that the sample data satisfied the core assumptions of ordered logistic regression. This confirmed the suitability of ordered categorical regression for investigating the factors affecting hospital economic compensation levels. The pseudo *R*-squared metrics for the model are: Cox and Snell *R^2^ = 0.044*, Nagelkerke *R^2^ = 0.048*, McFadden *R^2^ = 0.017*, Further analysis is presented in Table 5.

**Table 5:** The influencing factors of hospital economic compensation at the patient level.

		Estimate	S. E.	Wald	*p*	OR	95% Confidence Interval	
							Lower Bound	Upper Bound
Threshold	Hospital economic compensation level = 1	−1.394	0.158	78.174	0		−1.703	−1.085
	Hospital economic compensation level = 2	−0.041	0.15	0.076	0.783		−0.334	0.252
	Hospital economic compensation level = 3	2.024	0.184	121.108	0		1.663	2.384
	Hospital economic compensation level = 4	3.854	0.341	127.688	0		3.186	4.523
Location	Patient health impairment disability ratings	−0.225	0.057	15.475	0	0.8	−0.337	−0.113
	Dispute initiator = 1	0.169	0.212	0.634	0.426	1.18	−0.247	0.584
	Dispute initiator = 2	0a						
	Gender = 1	0.003	0.126	0.001	0.979	1	−0.243	0.25
	Gender = 2	0a						
	Risk attributes of doctor-patient relationship	−0.644	0.237	7.36	0.007	0.53	−1.11	−0.179
	Risk attributes of doctor-patient relationship	0a						

Table 5 shows that the disability level of injury has a significant negative impact on the amount of hospital economic compensation (*β = −0.225, p < 0.001*). The odds ratio (OR) was *0.80 (95% CI, 0.71–0.89)*, indicating that controlling for other variables, a one-level increase in disability severity reduces the odds of hospitals paying a higher level of compensation by 20%. The doctor-patient relationship risk attributes significantly negatively affected the amount of economic compensation (*β = −0.644, p = 0.007*). Compared to non-medical disturbance cases, medical disturbance cases resulted in a 47% significant reduction in the odds of hospitals paying a higher level of economic compensation (*OR = 0.53, 95% CI: 0.33–0.84, Ps = 0.007*). The dispute initiator (*β = 0.169, p = 0.426*) and patient gender (*β = 0.003, p = 0.979*) did not enter the final model, suggesting they indirectly influence hospital economic compensation level through other pathways.

To identify factors influencing dispute trials outcomes, a binary logistic regression analysis was conducted with dispute trials outcomes as the dependent variable, incorporating all patient-related independent variables and covariates. Model fit indices: *−2 Log Likelihood = 901.258, Cox & Snell R^2^ = 0.002*, *Nagelkerke R^2^ = 0.004*. The Hosmer-Lemeshow test indicates good model fit (*p > 0.05*). The results are presented in Table 6.

**Table 6:** Binary logistic regression analysis results of patient litigation outcomes.

	B	S. E.	Wald	*p*	OR
Dispute initiator (1)	−0.257	0.283	0.823	0.364	0.774
Gender (1)	−0.178	0.166	1.137	0.286	0.837
Patient health impairment disability ratings	0.043	0.076	0.326	0.568	1.044
Risk attributes of doctor-patient relationship (1)	−0.07	0.299	0.055	0.815	0.932
Constant	1.349	0.197	46.896	0	3.855

Table 6 indicates that the regression coefficients for all patient-related variables (dispute initiator, patient gender, disability level of health impairment, and risk attributes of the doctor-patient relationship) are statistically insignificant (Ps > 0.05). Therefore, the dispute initiator, patient gender, disability level of health impairment, and risk attributes of the doctor-patient relationship do not directly influence the dispute trials outcomes.

Combining the information from Tables 5, 6 reveals that the patient’s disability level due to health impairment and the risk attributes of the doctor-patient relationship are direct factors influencing the hospital economic compensation level. Meanwhile, the initiator of the dispute and the patient’s gender are indirect factors affecting the hospital economic compensation level.

## 5 Discussion

The results of the differential analysis indicate that the significant differences in the distribution of hospital economic compensation levels depending on the initiator of the dispute (*p < 0.001*), and Hypothesis 1 has been verified. This is consistent with research expectations: disputes initiated by patients themselves primarily seek to clarify the truth of diagnosis and treatment, with relatively moderate demands for financial compensation; whereas disputes initiated by non-patients (health stakeholders) in cases of patient death or severe disability focus more on compensating for economic losses, resulting in stronger demands and greater disparities in hospital economic compensation level (Sun et al., 2020). Comparing the risks in doctor-patient relationships arising from these two scenarios reveals distinct underlying drivers, naturally leading to noticeable differences in the frequency distribution of hospital economic compensation. This disparity vividly illustrates how differing psychological motivations and behavioral processes among dispute initiators lead to divergent outcomes in economic compensation. Of course, variations in economic investment due to differences in the diseases themselves cannot be ruled out as a potential contributing factor.

The results of the variance analysis indicated that the frequency distribution of hospital economic compensation levels did not differ significantly in terms of patient gender and doctor-patient relationship risk attributes. This finding suggests that the principle of gender equality is widely accepted in society (Williams et al., 2021), and the health rights of men and women are accorded equal importance. This is evident in the equal treatment of hospital economic compensation for health-related damages, resulting in no significant differences in the frequency distribution of compensation amounts based on patient gender. The lack of significant differences in case compensation amounts concerning doctor-patient relationship risk attributes, which indicates that judicial trials do not arbitrarily adjust the amount of financial compensation due to disturbances or unjustified interference from the patient or family. Of course, individual cases or data bias cannot be ruled out as potential influencing factors.

The results showed that the frequency distribution of dispute trials outcomes did not differ significantly across the four dimensions: dispute initiator, patient gender, disability ratings, and doctor-patient relationship risk attributes. This outcome reflects the impartiality of judicial decisions to a certain extent, which are not subjectively altered based on the dispute initiator’s purpose, patient gender, level of disability, or the risky behavior of the patient and family. Simultaneously, it should be cautiously interpreted that statistical insignificance merely indicates that no significant association was found for these patient-related variables examined in this study. Judicial adjudication of doctor-patient relationship risks focuses solely on the degree of health damage caused by medical actions to determine compensation rulings. In the context of doctor-patient relationship risk events, the court strictly examines the terms of justice to ensure a fair trial. The lack of significant differences in the frequency distribution of dispute trials outcomes is understandable, given the strict adherence to legal principles (Sun et al., 2021). Meanwhile, it must be acknowledged that this study cannot exhaustively account for all factors potentially influencing verdicts (such as sufficiency of evidence, attorney expertise, judicial bias, medical institution tier, media attention, or unobserved degrees of medical negligence). These unmeasured variables may exert influence on trial outcomes. The correlation analysis indicated that the hospital economic compensation was positively correlated with the outcome of the patient’s lawsuit, the initiator of the dispute, and the risk attributes of the doctor-patient relationship (Ps < 0.05), it was negatively correlated with the disability ratings (*p* < 0.01). The positive correlation with the hospital economic compensation is self-evident, as patients who win their lawsuits receive financial compensation for health losses, whereas those who lose do not. The positive correlation between the hospital economic compensation and the initiator of the dispute further corroborates the findings of the difference analysis, indicating that “the financial compensation pursued by patients and their family members is a key influencing factor.” One possible explanation for this is that in China, a higher disability ratings corresponds to less severe health damage, thereby resulting in lower financial compensation. In summary, it is reasonable to observe a negative correlation between the hospital economic compensation and the disability ratings. The positive correlation between the hospital economic compensation and the risk attributes of the doctor-patient relationship (*r = 0.082, p < 0.05*) may stem from the fact that patients often suffer health damage in medical activities. During the trial process, judges rule to provide patients and their families with appropriate financial compensation based on the principle that “it cannot be ruled out that the health damage is unrelated to the medical actions,” thereby reflecting societal compassion and humane care to mitigate medical disputes. This finding is consistent with our previous study, which showed that “patients’ emotions are related to the level of doctor-patient disputes” (Lu et al., 2024).

The correlation analysis revealed that the initiator of the dispute was negatively correlated with gender and disability ratings (*Ps < 0.01*), while gender was positively correlated with disability ratings (*p < 0.01*). The correlations between other variables were not statistically significant. These findings may be attributed to the greater family responsibilities of Chinese men compared to women, which lead to higher economic pressures and a greater incentive to initiate disputes over health damage. Specifically, a lower disability ratings, indicating more severe health damage, not only results in greater economic losses for the family but also directly interferes with the actions of health stakeholders, such as caregiving, thereby easily triggering disputes involving health stakeholders other than the patients themselves. In summary, these correlations can be explained by the interplay of family responsibilities, economic pressures, and the severity of health damage. The lack of significant correlations between other variables may reflect the impartiality of judicial trials in doctor-patient disputes, although individual cases or data biases cannot be ruled out as potential influencing factors.

Ordered regression analysis indicates that the disability level of injury exerts a significant negative impact on the amount of economic compensation paid by hospitals. This finding can be explained by the classification of disability levels and the valuation assigned to the degree of health impairment. The risk attributes of doctor-patient relationships exert a significant negative impact on the tier of economic compensation paid by hospitals. This suggests that China has begun to regulate and govern disruptive behavior in medical settings, a trend corroborated by the introduction of legal provisions addressing such conduct. The overall explanatory power assessment of the hospital economic compensation grading model revealed a low pseudo R-squared value (Nagelkerke R^2^ = 0.048). This hint that despite the statistical significance of the aforementioned factors, the patient-side variables included in this study collectively offer limited explanatory power for variations in hospital economic compensation. The determination of compensation amounts may remains be primarily influenced by legally mandated standards corresponding to disability grades, along with unobserved factors such as medical negligence and clinical contexts. Binary regression reveals that dispute initiator, patient gender, patient health impairment disability level, and medical-patient relationship risk attributes do not directly influence dispute trials outcomes. This finding is strongly supported by the model’s extremely low explanatory power (Nagelkerke R^2^ = 0.004). It indicates that judicial proceedings focus more on the nature of health impairment rather than the behavioral characteristics of the injured party. This indicates that judicial proceedings focus more on the essence of health impairment rather than patient behavioral characteristics. Of course, it’s not ruled out potential unmeasured variables (e.g., legal counsel quality, media attention) that could still influence rulings.

Convenience sampling resulted in incomplete coverage of the population. Although the selected sample size was large and subjectively validated by a professional legal team, selection bias may still exist since data originated solely from the China Judgments Online platform. Due to limitations in document information, key confounding variables such as the degree of medical negligence, disease type, and treatment complexity were not captured, potentially reducing the statistical power and precision of the analysis. Future research should employ more refined sampling methods, incorporate more diverse and objective variables, and utilize advanced econometric models for causal inference.

## 6 Conclusion

This empirical study demonstrates that within the scope of patient-related factors examined—dispute initiator, gender, risk attributes of the doctor-patient relationship, and degree of health impairment—no statistically significant association was found with judicial trial outcomes. The economic compensation that hospitals face due to doctor-patient relationship risks is directly influenced by the patient’s disability level and the attributes of the doctor-patient relationship risk and is indirectly affected by the initiator of the dispute and the patient’s gender. Therefore, we should optimize the risk management of medical disputes, focusing on improving doctor-patient communication, strengthening humanistic care, and guiding legal rights protection. Through rational interaction between doctors and patients, as well as fair judgments by judicial authorities, we can achieve harmonious and stable doctor-patient relationships.

## Data Availability

The original contributions presented in the study are included in the article/supplementary material, further inquiries can be directed to the corresponding authors.
